# Supplementary material for: Construction of an Ordered Transposon Library for Uropathogenic Proteus mirabilis HI4320
Source: Microbiol Spectr. 2022 Nov 15;10(6):e03142-22. doi: 10.1128/spectrum.03142-22 (PMC9769666; doi:10.1128/spectrum.03142-22)
Supplement: Supplemental file 1 — Fig. S1-S3. Download spectrum.03142-22-s0001.pdf, PDF file, 0.8 MB [file spectrum.03142-22-s0001.pdf]

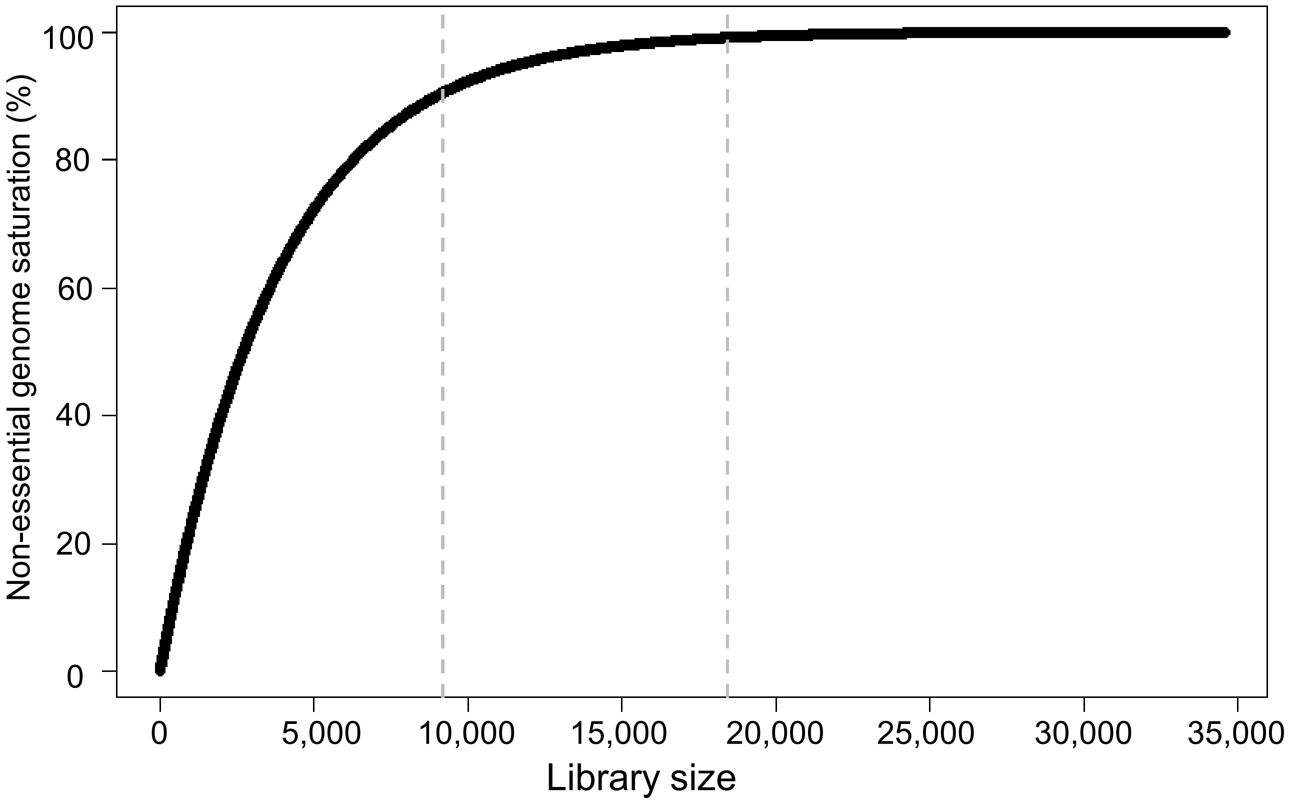

Supplemental Figure S1. **Model of genome saturation.** The 'coupon collector's' problem was used to generate a model of the library size required to generate transposon insertions in simulated non-essential genes. Using this model, one set of 96, 96-well plates containing 9,216 mutants would theoretically achieve 90.6% genome saturation and two sets of plates containing 18,432 mutants would mutate 99.1% of non-essential genome regions. The theoretical saturation for one or two sets of plates is shown as vertical gray dashed lines.

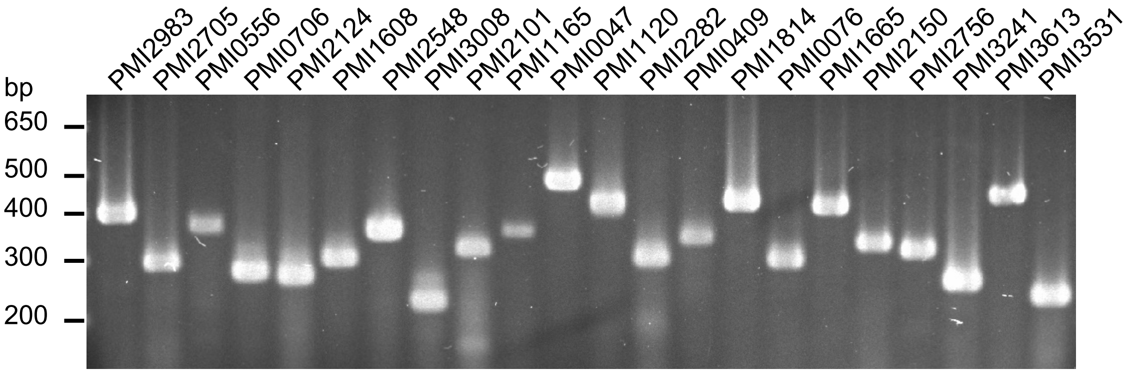

Supplemental Fig. S2. Validation of transposon position calls by anchored PCR. PCR was used to confirm transposon insertion in 25 randomly selected mutants using one transposon-specific anchored primer and one gene-specific primer. 22/25 mutants were confirmed to have a transposon in the predicted location (not shown: the three PCR-negative mutants). Size markers shown on left.

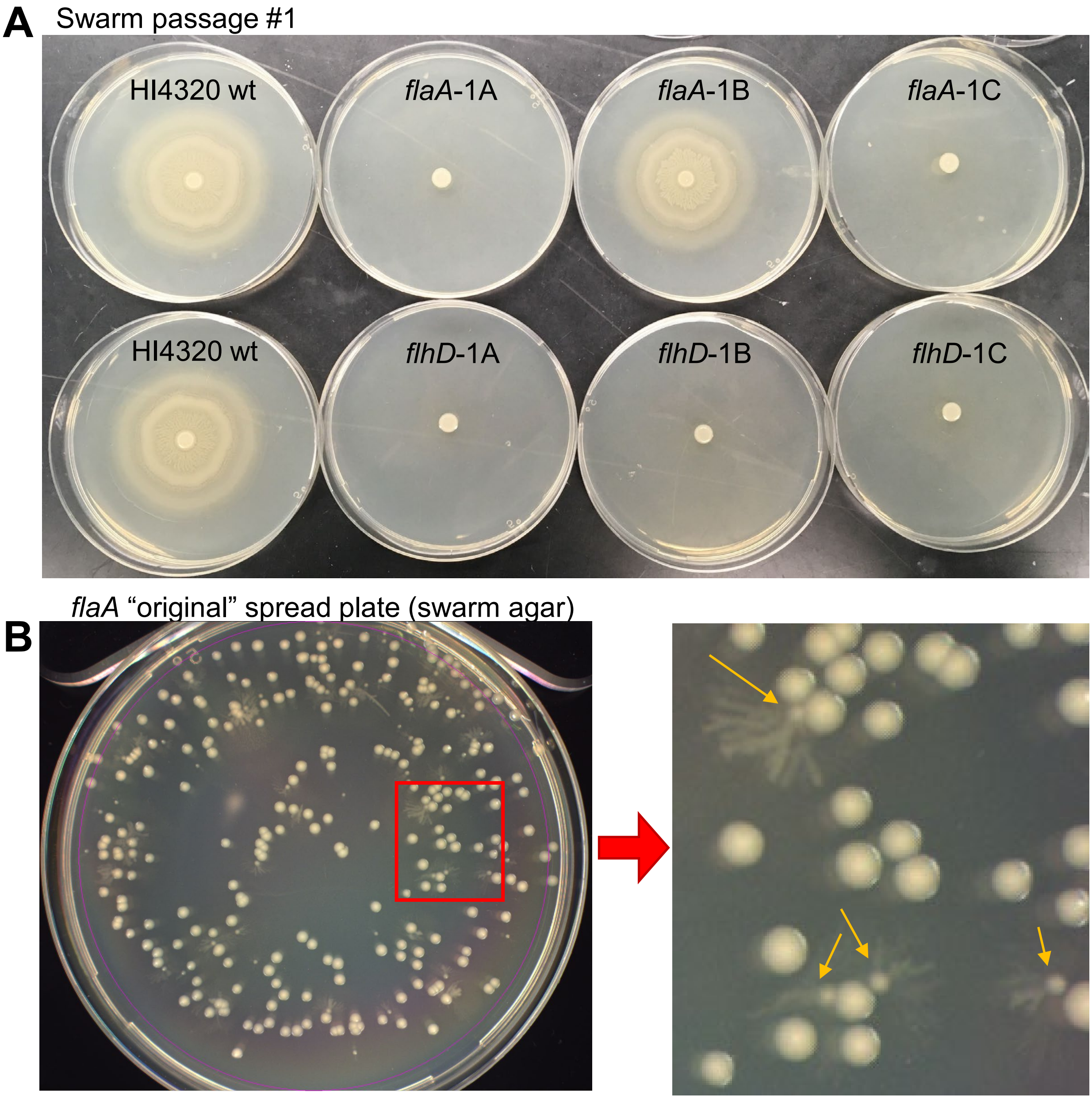

Supplemental Fig. S3. Swarming motility by *flhD* and *flaA* mutants. **(A)** Ordered library mutants predicted to contain transposon insertions in flagellar genes *flhD* or *flaA* were passaged both in broth and on agar and observed for restoration of swarming motility. The *flhD* mutant remained nonmotile throughout the duration of the experiment; the first passage of three colonies is shown here. Passage of the *flaA* mutant resulted in both motile (*flaA*-1B) and nonmotile variants (*flaA*-1A and 1C). Motile isolates, such as *flaA*-1B, showed two transposon insertions on Southern blots (**Fig. 3**, lane 4). Nonmotile isolates, such as *flaA*-1A, showed a single transposon insertion (**Fig. 3**, lane 3). **(B)** Following the observation of motile “*flaA*” derivatives, the primary streak of the “*flaA*” well contents was cultured and dilution-plated to obtain single colonies on swarm agar, resulting in a mixture of swarming and non-swarming colonies. Notably, the swarming colonies, denoted with orange arrows in the inset image, appeared smaller than the nonmotile colonies in this assay. A non-swarming colony was cultured to generate the sample in **Fig. 3**, lane 5 (one band). A swarming colony was similarly cultured to generate the sample in **Fig. 3**, lane 6 (two bands).
